# The Role of the *Exo-Xis* Region in Oxidative Stress-Mediated Induction of Shiga Toxin-Converting Prophages

**DOI:** 10.1155/2016/8453135

**Published:** 2015-12-20

**Authors:** Katarzyna Licznerska, Aleksandra Dydecka, Sylwia Bloch, Gracja Topka, Bożena Nejman-Faleńczyk, Alicja Węgrzyn, Grzegorz Węgrzyn

**Affiliations:** ^1^Department of Molecular Biology, University of Gdańsk, Wita Stwosza 59, 80-308 Gdańsk, Poland; ^2^Laboratory of Molecular Biology (Affiliated with the University of Gdańsk), Institute of Biochemistry and Biophysics, Polish Academy of Sciences, Wita Stwosza 59, 80-308 Gdańsk, Poland

## Abstract

Previous studies indicated that these genetic elements could be involved in the regulation of lysogenization and prophage induction processes. The effects were dramatic in Shiga toxin-converting phage Φ24_B_ after treatment with oxidative stress-inducing agent, hydrogen peroxide, while they were less pronounced in bacteriophage *λ* and in both phages irradiated with UV. The hydrogen peroxide-caused prophage induction was found to be RecA-dependent. Importantly, in hydrogen peroxide-treated *E. coli* cells lysogenic for either *λ* or Φ24_B_, deletion of the *exo-xis* region resulted in a significant decrease in the levels of expression of the S.O.S. regulon genes. Moreover, under these conditions, a dramatic decrease in the levels of expression of phage genes crucial for lytic development (particularly *xis, exo, N, cro, O, Q*, and *R*) could be observed in Φ24_B_-, but not in *λ*-bearing cells. We conclude that genes located in the *exo-xis* region are necessary for efficient expression of both host S.O.S regulon in lysogenic bacteria and regulatory genes of Shiga toxin-converting bacteriophage Φ24_B_.

## 1. Introduction

Infection of humans by enterohemorrhagic* Escherichia coli* (EHEC) strains causes hemorrhagic colitis, and in some patients it may result in various complications, including, the most severe of them, the hemolytic-uremic syndrome and neurological dysfunctions [1–3]. The main causes of EHEC-mediated complications are Shiga toxins, produced by the infecting bacteria [4]. The severity of EHEC infections and significance of the medical problem related to them are exemplified by local outbreaks, occurring in various geographical regions around the world. One of the most famous of them took place in 2011 in Germany, where over 4,000 patients developed severe symptoms, and 54 died [5–10].

In EHEC strains, Shiga toxins are encoded by genes (called* stx* genes) located in genomes of prophages [11, 12]. The phages bearing* stx* genes are referred to as Shiga toxin-converting bacteriophages, and all of them belong to the family of lambdoid phages (with phage *λ* serving as a paradigm) [12].* stx* genes are present between *Q* antiterminator gene and the genes coding for proteins causing cell lysis; thus, in the lysogenic state, these genes are not transcribed [13–15] and Shiga toxins are not produced. Their expression is possible only after prophage induction [11, 12] which usually requires activation of the bacterial S.O.S. response, mediated by RecA protein, though RecA-independent induction of Shiga toxin-converting prophages by EDTA has also been reported [16].

During infection of human intestine by EHEC, the oxidative stress appears to be the most likely condition causing the bacterial S.O.S. response and subsequent induction of Shiga toxin-converting prophages [17]. In fact, it was demonstrated that hydrogen peroxide (which is produced by neutrophils as a response to infection) enhanced production of Shiga toxins by EHEC [18] due to oxidative stress-mediated induction of Shiga toxin-converting prophages [19, 20].

**Figure 1:**
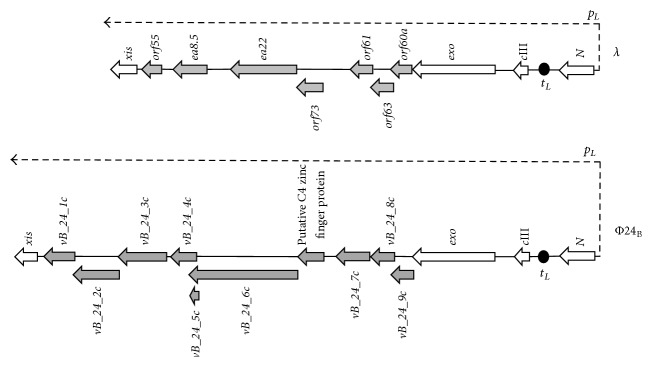
Schematic maps of the* exo-xis* regions of bacteriophages *λ* and Φ24_B_. Genes from the* exo-xis* region are marked as thick grey arrows, and other genes are shown as thick open arrows. Directionality of transcription from *p*_*L*_ promoter is indicated as thin dashed arrow. *t*_*L*1_ terminator is marked as black oval.

Since many antibiotics not only kill bacteria and inhibit their growth but also induce prophage lytic development, their use is not recommended when EHEC infection is confirmed or even suspected (reviewed in [12]). Therefore, there are serious problems with treatment of patients, indicating that searching for new targets of potential therapies against Shiga toxin-producing bacteria is important. One might consider that such therapies should be focused on inhibition of Shiga toxin-converting prophage induction which would impair production of the EHEC virulence factor. All lambdoid phages, including Shiga toxin-converting bacteriophages, contain the *b* region in their genomes which is dispensable for the development under standard laboratory conditions [14, 15]. Inside this part of the phage genome, there is an evolutionarily conserved fragment, located between* exo* and* xis* genes and transcribed from *p*_*L*_ promoter, called the* exo-xis* region (Figure 1). This region encompasses several genes and open reading frames whose functions in phage development are largely unknown, and only a few articles are available in the literature that focused on them. Nevertheless, some interesting observations have been reported. Namely, induction of expression of genes from the* exo*-*xis* region resulted in synchronization of the host cell cycle [21] and inhibition of host DNA replication [22]. Moreover, overexpression of these genes impaired lysogenization of* E. coli* by bacteriophage *λ* [23] and enhanced induction of prophages *λ* and Φ24_B_ (one of Shiga toxin-converting phages) [24]. Ea8.5 protein, encoded by a gene located in the* exo-xis* region, contains a fused homeodomain/zinc finger fold [25] which suggests a regulatory role for this protein. Interestingly, prophage induction with mitomycin C or hydrogen peroxide caused different expression patterns of genes from the* exo-xis* region; such differences were observed in both phages, *λ* and Φ24_B_ [26]. In this work, we used the deletion mutants to investigate the role of the* exo-xis* region in induction of *λ* and Φ24_B_ prophages under oxidative stress conditions.

## 2. Materials and Methods

### 2.1. Bacteria and Bacteriophages

*E. coli* MG1655 strain [27] and its derivatives, used in this work, are listed in Table 1. Bacteria were routinely cultured in the Luria-Bertani (LB) medium at 30°C (most experiments) or 37°C (lysogenization and recombination procedures during construction of strains and SOS ChromoTest, according to the instructions of kits' manufacturers), under aerobic conditions. Where appropriate, the following antibiotics were added: chloramphenicol up to 20 *μ*g/mL, kanamycin up to 50 *μ*g/mL, and/or tetracycline up to 12.5 *μ*g/mL.

Bacteriophages *λ* papa (from our collection) [26] and Φ24_B_ (Δ*stx2*::*cat*) [28] were employed in this study. Phage suspensions were stored in the TM buffer (10 mM Tris-HCl, 10 mM MgSO_4_, pH 7.2) at 4°C.

The deletion mutants were constructed as described previously [29], by using the Quick and Easy* E. coli* Gene Deletion Kit (from Gene Bridges). The deletion of the indicated region was performed according to the manufacturer's protocol using primers listed in Table 2. In the first step, the targeted sequence has been replaced with the FRT-flanked kanamycin resistance cassette, and the selection marker was subsequently removed in the FLP-recombinase step, leaving only 87 nucleotides of the cassette in the place of the original sequence. Each deletion was confirmed by DNA sequencing.

Lysogenic strains were constructed according to the procedure described previously [24], with slight modifications. Briefly, host bacteria were cultured to A_600_ of 0.5 in LB medium supplemented with MgSO_4_ and CaCl_2_ (to final concentrations of 10 mM each) at 37°C with shaking. At this point, one milliliter of the culture was withdrawn and centrifuged (10 min, 2000 ×g). Pellet was washed twice with TCM buffer (10 mM Tris-HCl, pH 7.2, 10 mM MgSO_4_, 10 mM CaCl_2_) and then suspended in 1 mL of the same buffer. Next, bacteria were incubated for 30 min at 30°C and mixed with phage suspensions at multiplicity of infection (m.o.i.) = 5. Mixtures of bacteria and phages were incubated in TMC buffer for 30 min at 30°C; then serial dilutions were prepared in TM buffer (10 mM Tris-HCl, 10 mM MgSO_4_; pH 7.2), and the mixture was plated onto LB agar. Plates were incubated at 37°C overnight. Lysogens were verified by sensitivity to UV irradiation and confirmed by PCR with primers designed to amplify phage sequence (Table 3).

### 2.2. Phage Lytic Development after Prophage Induction

Bacteria lysogenic with tested phages were cultured in LB medium at 30°C to A_600_ of 0.1. Two induction agents were tested: H_2_O_2_ (1 mM) and UV irradiation (50 J/m^2^; this dose was achieved by 20 sec incubation of the bacterial suspensions in Petri dishes under UV lamp hanged 17 cm above the laboratory table). At indicated times after induction, samples of bacterial cultures were harvested, and 30 *μ*L of chloroform was added to 0.5 mL of each sample. The mixture was vortexed and centrifuged for 5 min in a microcentrifuge. Then, serial dilutions were prepared in TM buffer, and phage titer (number of phages per mL) was determined by spotting 2.5 *μ*L of each dilution of the phage lysate on a freshly prepared LB agar (1.5%) or LB agar with 2.5 *μ*g/mL chloramphenicol (according to a procedure described previously [30]), with a poured mixture of 1 mL of the indicator* E. coli* MG1655 strain culture and 2 mL of 0.7% nutrient agar (prewarmed to 45°C), supplemented with MgSO_4_ and CaCl_2_ (to a final concentration of 10 mM each). When full-plate titration was used, 0.1 mL of phage lysate dilutions was plated onto LB agar. Plates were incubated at 37°C overnight. Analogous experiment but without induction agents (control experiments), which allows estimation of effects of spontaneous prophage induction, was performed with each lysogenic strain. The relative phage titer, expressed as plaque forming units (pfu)/mL, was calculated by subtracting the values obtained in the control experiment from the values determined in the main experiment, and as a consequence it represents the ratio of phage titers in induced and noninduced cultures. Each experiment was repeated three times.

### 2.3. The S.O.S. Assay

The S.O.S. assay was performed using the SOS-ChromoTest Kit (Environmental Bio-Detection Products Inc.), following the manufacturer's protocol and using provided 4-nitro-quinoline oxide (4-NQO) as a positive reference standard, and 1 mM H_2_O_2_ and UV irradiation (50 J/m^2^) as tested inducers of the S.O.S. response [31, 32]. In the case of UV light irradiation, the production of *β*-galactosidase was evaluated immediately after the exposure, without 2 h incubation at 37°C (recommended by the manufacturer), as, without this modification, the visual detection of the blue color was not possible due to rapid S.O.S. response after UV irradiation. Before use, the SOS-ChromoTest bacterial strain (*E. coli* PQ37, provided with the kit) was lysogenized by following phages: *λ*, *λ*Δ*exo-xis*, Φ24_B_, or Φ24_B_Δ*exo-xis*, according to procedure described above.

### 2.4. Preparation of RNA and cDNA from Bacteria

For the isolation of total RNA, the previously described [26] procedure was employed. Briefly, the prophage induction was performed with 1 mM H_2_O_2_ or UV irradiation (at the dose of 50 J/m^2^). Following induction, the samples were withdrawn at indicated times and the growth of bacteria was inhibited by the addition of NaN_3_ (Sigma-Aldrich) to a final concentration of 10 mM. Total RNA was isolated from 10^9^ bacterial cells by using the High Pure RNA Isolation Kit (Roche Applied Science). Bacterial genomic DNA carryover was removed by incubation with TURBO DNase from TURBO DNA-*free* Kit (Life Technologies) for 60 min at 37°C, according to the manufacturer's guidelines. To evaluate the quality and quantity of the isolated RNA, a NanoDrop spectrophotometer was employed, considering the absorbance ratio (which should be 1.8 ≤ A260/A280 ≤ 2.0). Moreover, band patterns of total RNA were visualized by electrophoresis. The absence of DNA from RNA samples was controlled by PCR amplification and by real-time PCR amplification (all analyzed genes were tested). RNA preparations were stored at −80°C. cDNA was obtained with Transcriptor Reverse Transcriptase and random hexamer primers (Roche Applied Science), using total RNA samples (1.25 *μ*g) as templates. cDNA reaction mixtures were diluted 10-fold for use in real-time PCR.

### 2.5. Real-Time PCR Assay and Data Analysis

The patterns of genes' expression were determined by quantitative real-time reverse transcription-PCR (qRT-PCR), using the LightCycler 480 Real-Time PCR System (Roche Applied Science) and cDNA samples from lysogenic bacteria. Transcripts of tested phage and bacterial genes were compared in parallel to* 16S rRNA* housekeeping gene (according to a procedure described previously [34]), whose expression was found to be constant. Primers were developed by Primer3web version 4.0.0 and produced by Sigma-Aldrich or GENOMED. The transcriptional analysis of phage and bacterial genes from lysogenic strains was performed with primers presented in Table 3. Real-time PCR amplifications were carried out for 55 cycles in 20 *μ*L reaction volume, using LightCycler 480 SYBR Green I Master (Roche Applied Science) as a fluorescent detection dye. Reactions were performed in Roche 96-well plates containing 10 *μ*L 2x SYBR Green I Master Mix, 6.25 ng/*μ*L cDNA, and 200 nM of each gene-specific primer (Table 3). Relative quantification assays were performed with cDNA of 16S rRNA and phage/bacterial genes multiplex assay. All templates were amplified using the following program: incubation at 95°C for 5 min, followed by 55 cycles of 95°C for 10 s, 60°C for 15 s, and 72°C for 15 s. No template control was included with each run. The specificity of amplified products was examined by melting curve analysis immediately after the final PCR cycle and confirmed by gel electrophoresis. Each experiment was conducted in triplicate.

The relative changes in gene expression revealed by quantitative real-time PCR experiments were analyzed using the calibrator, normalizing relative quantification method with efficiency correction (called the E-Method). This method has been used and described in detail previously [26, 29, 35]. The values obtained at time 0 (representing the conditions of spontaneous prophage induction) were used as calibrators. Thus, the following equation was employed to calculate the final results:

(1) Normalized  relative  ratio=EtCTt  calibrator−CTt  sampleErCTr  calibrator−CTr  sample,

where *t* is target and *r* is reference.

### 2.6. In Silico Analyses

The multiple sequence alignment was performed using the ClustalW algorithm available at the website http://www.genome.jp/tools/clustalw/. The Pfam protein families database [36], available at the website http://pfam.xfam.org/, was used to identify protein domains.

## 3. Results and Discussion

### 3.1. Deletion of the* Exo-Xis* Region Impairs Φ24_B_ but Not *λ* Prophage Induction after Treatment with Hydrogen Peroxide

**Figure 2:**
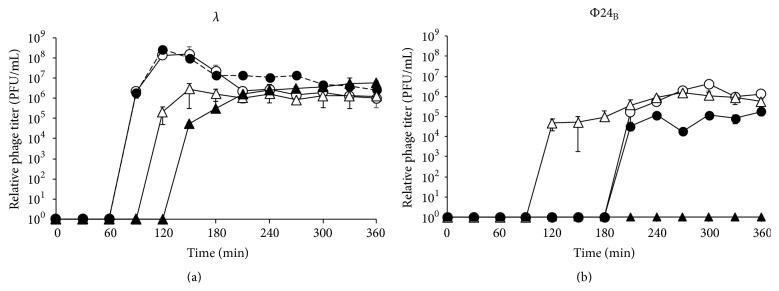
Lytic development of bacteriophages *λ* (a) and Φ24_B_ (b), either wild-type (open symbols) or Δ*exo-xis* (closed symbols), after induction of lysogenic* E. coli* MG1655 with UV irradiation (50 J/m^2^, circles) or hydrogen peroxide (1 mM, triangles). The presented results are mean values from 3 independent experiments (biological samples), with error bars indicating SD. Statistically significant differences (*p* < 0.05 in* t*-test) between wild-type and Δ*exo-xis* phages were found at times 270, 300, and 360 min of experiments with hydrogen peroxide and at 270, 300, 330, and 360 min of experiments with UV for *λ* and at times 120, 240, 270, 300, 330, and 360 min of experiments with hydrogen peroxide and at 270, 300, 330, and 360 min of experiments with UV for Φ24_B_.

Until now, all* in vivo* studies on effects of the* exo-xis* region on host or phage development were performed with the use of strains overexpressing genes from this region [21–24, 26]. In this work, we have constructed a series of bacteriophage *λ* and Φ24_B_ mutants with deletions of either the whole* exo-xis* region or individual genes or open reading frames (Table 1). When wild-type *λ* and Φ24_B_ prophages were induced by UV irradiation (employed in this work as positive control conditions causing effective prophage induction) or hydrogen peroxide treatment of the lysogenic cells, efficiencies of induction and further phage lytic development were comparable in both phages, though some differences were observed in the duration of the lag phase of the phage development (Figure 2). Induction of *λ*Δ*exo-xis* mutant with UV irradiation was similar to that observed for the wild-type *λ*, and treatment with hydrogen peroxide caused only a slight delay in the mutant phage development. The decrease in the phage titer at later times of the experiments is characteristic for *λ* and most probably arises from adsorption of the progeny virions on fragments of disrupted cell envelopes [15, 24]. However, induction of Φ24_B_Δ*exo-xis* prophage by UV irradiation was less efficient than that of the wild-type Φ24_B_, and induction of the mutant by hydrogen peroxide was severely impaired (Figure 2). More detailed analyses, based on the full-plate phage titration method, allowing detection of 10 pfu/mL (see Section 2 for details), indicated that the number of pfu per mL of Φ24_B_Δ*exo-xis* phage after induction with hydrogen peroxide was at the same range (10^3^/mL) as that measured without specific induction (i.e., representing efficiency of spontaneous prophage induction). Nevertheless, the titer of Φ24_B_Δ*exo-xis* measured at 240 and 360 min after induction was 9.0 ± 0.2 × 10^3^ and 7.9 ± 0.9 × 10^3^, respectively, that is, still 3-4 times higher than that without induction, which was 2.1 ± 0.3 × 10^3^ and 2.6 ± 0.4 × 10^3^, respectively (note that the titer of the wild-type Φ24_B_ after prophage induction was several orders of magnitude higher than that without induction, Figure 2).

Deletions of individual genes and open reading frames from the* exo-xis* region in Φ24_B_ did not affect significantly the phage titer. However, such deletions resulted in delays in prophage induction by hydrogen peroxide (Table 4). Interestingly, when prophage induction was stimulated by UV irradiation, such effect was not observed, and in some cases even more rapid induction of the mutant prophages occurred. In bacteriophage *λ*, only slight effects of deletions of individual genes and open reading frames were detected (Table 4).

We conclude that the genes and open reading frames from the* exo-xis* region play important roles in the regulation of lambdoid prophage induction, as deletions of the whole region or single* loci* caused significant changes in efficiency and timing of this process. The effects of mutations are more pronounced in Shiga toxin-converting phage Φ24_B_ than in *λ* and in lysogenic* E. coli* cells treated with hydrogen peroxide than in UV-irradiated ones. Thus, the* exo-xis* region seems to be particularly important for Φ24_B_ phage under conditions of the oxidative stress, the most likely conditions causing Shiga toxin-converting prophage induction during infection with EHEC.

### 3.2. Hydrogen Peroxide-Mediated Prophage Induction Is a RecA-Dependent Process

**Figure 3:**
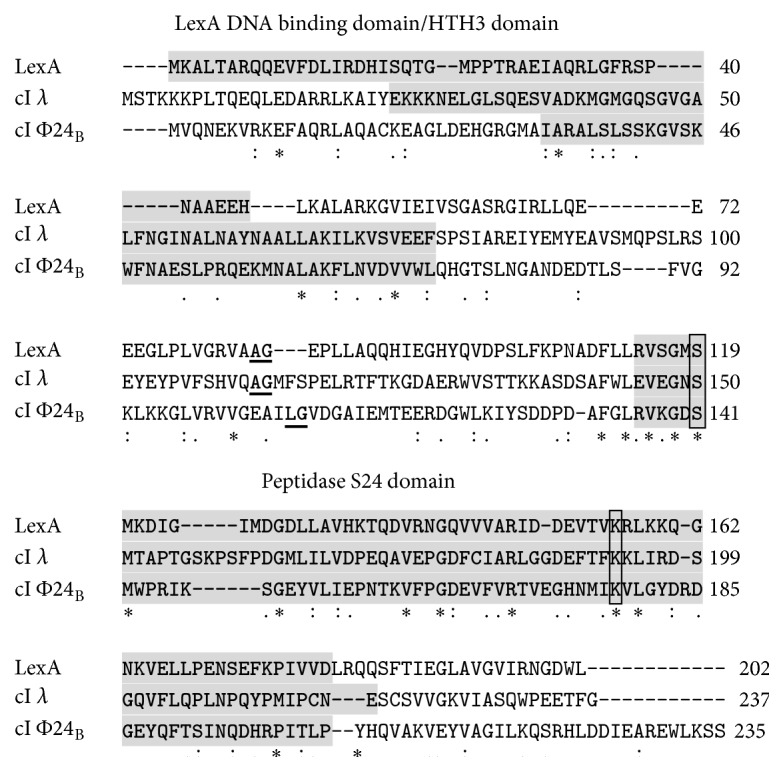
Alignment of amino acid sequences of* E. coli* LexA protein and cI repressors of bacteriophages *λ* and Φ24_B_. Specific protein domains are indicated by grey background. Self-cleavage sites are underlined (two amino acid residues between which the cleavage occurs). The active sites of the peptidase domains are framed. Symbols under the sequence alignment indicate conserved sequence (*∗*), conservative mutations (:), semiconservative mutations (.), and nonconservative mutations ().

Efficient induction of lambdoid prophages is a RecA-dependent process due to a molecular mimicry between the phage cI repressor and the host-encoded LexA repressor which is self-cleaved after stimulation by the activated form of RecA protein under the S.O.S. response conditions [12–15]. Such a mimicry is well known for bacteriophage *λ* cI protein and LexA [12, 13], and we found that both domain structure and amino acid residues crucial for the self-cleavage are also conserved in cI repressor of phage 24_B_ (Figure 3) (note that cI sequence of 24_B_ is identical to that of another Shiga toxin-converting bacteriophage, 933 W [37]). Nevertheless, since RecA-independent induction of Shiga toxin-converting prophages has also been reported [16], we asked whether hydrogen peroxide-caused prophage induction depends on the activation of the S.O.S. response.

**Figure 4:**
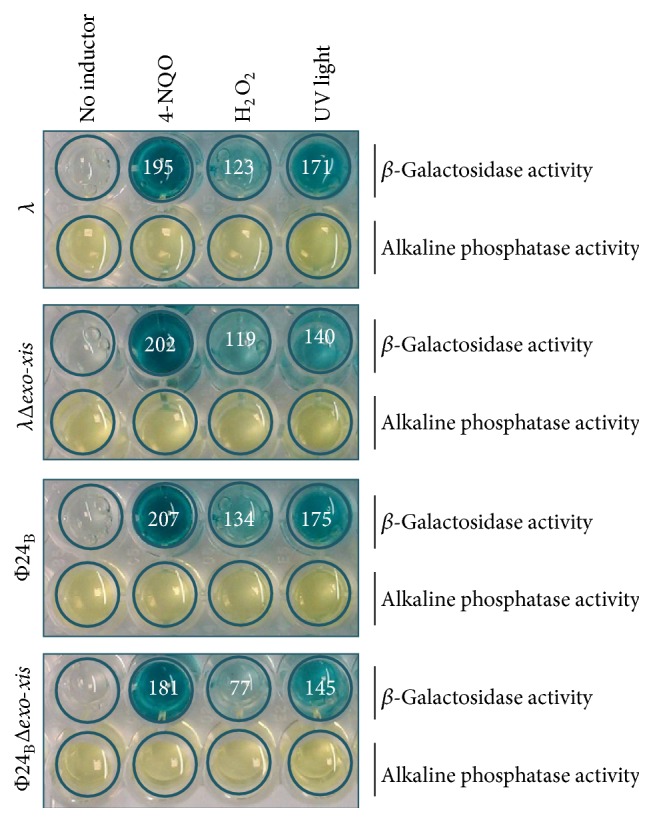
Induction of the S.O.S. response in* E. coli* PQ37 lysogenic with *λ*, *λ*Δ*exo-xis*, Φ24_B_, or Φ24_B_Δ*exo-xis*, treated with 4-NQO (4-nitro-quinoline oxide, positive control), H_2_O_2_ (1 mM), or UV (50 J/m^2^), using the SOS ChromoTest. *β*-Galactosidase activity (identified by the blue spots) represents induction of the S.O.S. regulon. Alkaline phosphatase activity (identified by yellow spots) evaluates viability of tested bacteria. Quantification of *β*-galactosidase activity was performed by densitometry, using the ImageJ software (available at http://imagej.nih.gov/ij/index.html). The results (in arbitrary units reflecting value = 1 ascribed to samples with no inductor), presented as numbers inside the corresponding spots, are mean values from three measurements (with SD < 10% in each case). All these values were significantly (*p* < 0.001 in* t*-test) higher than that in the control experiments with no inductor. When Δ*exo-xis* mutants were compared to wild-type phages, the only significant difference (*p* < 0.05 in* t*-test) occurred between Φ24_B_ and Φ24_B_Δ*exo-xis* lysogens induced with hydrogen peroxide.

When testing H_2_O_2_- or UV-dependent induction of prophages *λ*, *λ*Δ*exo-xis*, Φ24_B_, and Φ24_B_Δ*exo-xis* in* recA13* mutant host, in the assays analogous to those presented in Figure 2, pfu/mL values were at the levels of those estimated for the uninduced controls (1.8 ± 0.1 × 10^2^, 4.1 ± 1.4 × 10^2^, 1.3 ± 0.1 × 10^3^, and 2.8 ± 0.3 × 10^3^ pfu/mL for *λ*, *λ*Δ*exo-xis*, Φ24_B_, and Φ24_B_Δ*exo-xis*, resp.). Therefore, we conclude that induction of the investigated prophages under conditions of the oxidative stress (treatment with hydrogen peroxide) strongly depends on RecA function. Indeed, in cells lysogenic for *λ* or Φ24_B_ and treated with UV light or hydrogen peroxide, efficient induction of the S.O.S. response was evident, as estimated with the SOS ChromoTest (Figure 4). Intriguingly, while induction of the S.O.S. response by hydrogen peroxide in *λ*Δ*exo-xis* lysogen was comparable to that in *λ* lysogen, the signal in the SOS ChromoTest in Φ24_B_Δ*exo-xis* lysogen was considerably weaker than in the analogous experiment with Φ24_B_ lysogen (Figure 4). No such difference could be observed in UV-irradiated bacteria (Figure 4).

### 3.3. Deletion of the* Exo-Xis* Region Negatively Influences Expression of Genes from the S.O.S. Regulon in Hydrogen Peroxide-Treated Lysogenic Bacteria

**Figure 5:**
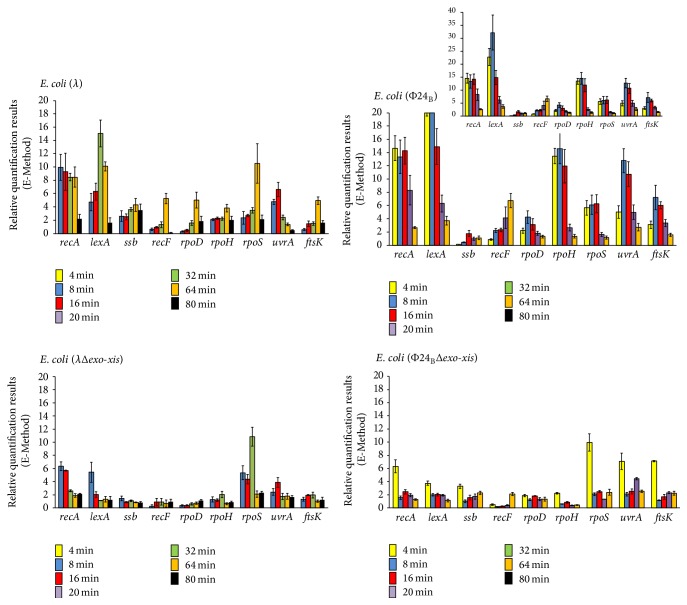
Expression of genes from the S.O.S. regulon in* E. coli* MG1655 lysogenic with *λ*, *λ*Δ*exo-xis*, Φ24_B_, or Φ24_B_Δ*exo-xis*, at indicated times after treatment with 1 mM H_2_O_2_, as estimated by reverse transcription quantitative real-time PCR. The values obtained with untreated cells were used as calibrators and were subtracted from the values determined at particular time points; thus, the presented values indicate the induction of expression of tested genes. The presented results are mean values from 3 independent experiments (biological samples), with error bars indicating SD. The additional panel for* E. coli* (24_B_) represents the results with different scale.

Since unexpected results were obtained in experiments with hydrogen peroxide-treated Φ24_B_Δ*exo-xis* lysogenic cells (Figure 4), we aimed to investigate the phenomenon of a less efficient induction of the S.O.S. response in more detail. Thus, expression of genes from the S.O.S. regulon was tested by reverse transcription quantitative real-time PCR in* E. coli* cells lysogenic for *λ*, *λ*Δ*exo-xis*, Φ24_B_, and Φ24_B_Δ*exo-xis* and treated with hydrogen peroxide. In both *λ* and Φ24_B_, deletion of the* exo-xis* region caused a significant reduction in the mRNA levels of most of the S.O.S. regulon genes relative to wild-type prophages, with exceptions of* rpoS* gene in both phages and* ssb, uvrA*, and* ftsK* genes in Φ24_B_, especially at later times after the treatment (Figure 5). Interestingly, in the case of wild-type Φ24_B_ lysogenic cells, the enhanced expression of particular genes from the S.O.S. regulon persisted longer, in most cases until 16 min after induction, whereas in the deletion mutant it decreases after 4 min (Figure 5). The impairment in expression of genes from the S.O.S. regulon (in particular* recA* and* lexA* genes, encoding the main regulators of the S.O.S. response) in the absence of the* exo-xis* region was more pronounced in Φ24_B_ than in *λ*. Moreover, induction of the S.O.S. regulon occurred significantly earlier in Φ24_B_ and Φ24_B_Δ*exo-xis* lysogens than in cells bearing *λ* and *λ*Δ*exo-xis* prophages (Figure 5). These results might explain, at least partially, effects of deletions of* exo-xis* genes on prophage induction, demonstrated in Figure 2 and Table 4, particularly delayed induction of Φ24_B_ prophage devoid of certain genes and open reading frames, and less pronounced effects of their lack in *λ* than in Φ24_B_.

Indications that overexpression of some genes from the* exo-xis* region of *λ* can influence host cell cycle and DNA replication have been reported previously [21, 22]. Suggestions that some genes of Φ24_B_ prophage may affect host growth were also published [38]. However, the results described in this subsection demonstrate for the first time that the* exo-xis* region can significantly modulate one of global cellular responses, the S.O.S. response, after treatment with hydrogen peroxide.

### 3.4. Expression of Crucial Phage Genes Is Dramatically Decreased after Treatment of Lysogenic Cells with Hydrogen Peroxide in the Absence of the* Exo-Xis* Region in Φ24_B_ Prophage

**Figure 6:**
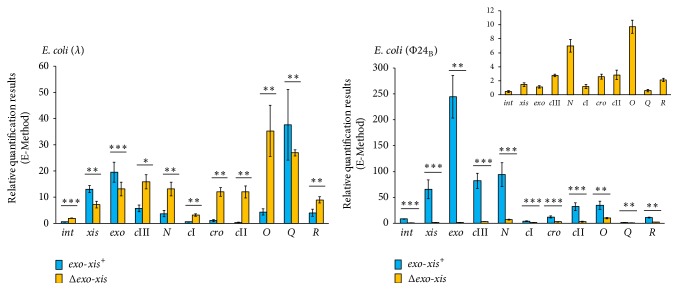
Expression of selected bacteriophage genes in* E. coli* MG1655 lysogenic with *λ* or Φ24_B_, either wild-type (blue columns) or Δ*exo-xis* (yellow columns) at 160 min after treatment with 1 mM H_2_O_2_, as estimated by reverse transcription quantitative real-time PCR. The values obtained with untreated cells were used as calibrators and were subtracted from the values determined at particular time points; thus, the presented values indicate the induction of expression of tested genes. The presented results are mean values from 3 independent experiments (biological samples), with error bars indicating SD. The additional panel for* E. coli* (24_B_) represents the results of Δ*exo-xis* variant with different scale, due to very small values measured. Statistically significant differences (in* t*-test) are marked as follows: ^*∗*^ *p* < 0.05, ^*∗∗*^ *p* < 0.01, and ^*∗∗∗*^ *p* < 0.001.

Expression of phage genes, crucial for the regulatory processes and lytic development, has been tested under the same conditions as described in the preceding subsection. The specific conditions and time after addition of hydrogen peroxide into the cell culture at which samples were withdrawn were chosen on the basis of similar experiments reported previously [26]. Interestingly, different effects of the deletion of the* exo-xis* region were observed for phages *λ* and 24_B_. In *λ*, deletion of genes and open reading frames located between* exo* and* xis* genes did not cause considerable effects on mRNA levels for* xis, exo*, and* Q*, whereas expression of* int, c*III,* N, c*I*, cro, c*II,* O*, and* R* was enhanced upon treatment with hydrogen peroxide (Figure 6). Completely different results were obtained when Φ24_B_ and Φ24_B_Δ*exo-xis* lysogens were studied; namely, expression of all tested genes was drastically impaired in hydrogen peroxide-treated bacteria in the absence of the* exo-xis* region on the prophage (Figure 6).

While negative regulation of transcription from cII-dependent promoters by overexpression of the* exo-xis* region has been reported previously in phage *λ* [23], this study demonstrated for the first time significant effects of this region on expression of a battery of phage genes under conditions of the oxidative stress. The results presented in Figure 6 for phage *λ* are compatible with those published previously (though obtained with different methods) [23], as overexpression of the* exo-xis* region had opposite effects to those observed in its absence. On the other hand, severely impaired expression of all tested phage genes in Φ24_B_Δ*exo-xis* was unexpected. However, these results (Figure 6) can explain a strong defect in the induction of Φ24_B_Δ*exo-xis* prophage (and perhaps further lytic development) by hydrogen peroxide (Figure 2). Similarly, drastic differences between effects of Δ*exo-xis* mutations on hydrogen peroxide-mediated prophage induction between *λ* and 24_B_ (Figure 2) can be ascribed to opposite regulation of expression of phage genes in the absence of the* exo-xis* region.

### 3.5. Effects of the* Exo-Xis* Region on Expression of Host and Phage Gene in UV-Irradiated Lysogenic Cells

**Figure 7:**
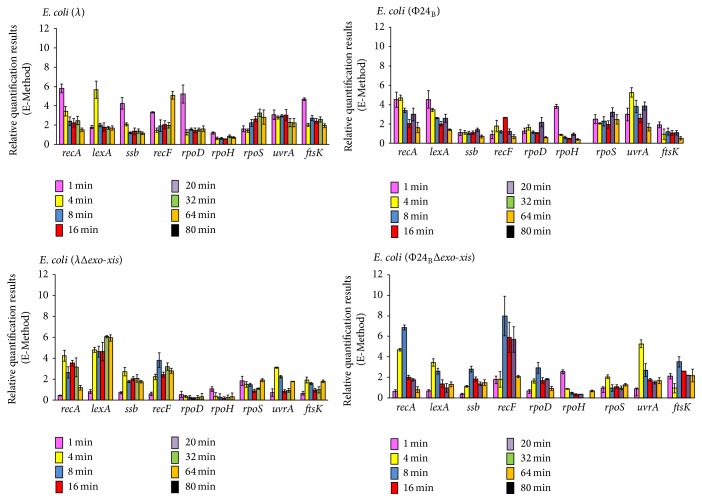
Expression of genes from the S.O.S. regulon in* E. coli* MG1655 lysogenic with *λ*, *λ*Δ*exo-xis*, Φ24_B_, or Φ24_B_Δ*exo-xis*, at indicated times after UV irradiation (50 J/m^2^), as estimated by reverse transcription quantitative real-time PCR. The values obtained with untreated cells were used as calibrators and were subtracted from the values determined at particular time points; thus, the presented values indicate the induction of expression of tested genes. The presented results are mean values from 3 independent experiments (biological samples), with error bars indicating SD.

Experiments analogous to those described in two preceding subsections were performed with lysogenic cells irradiated with UV. Interestingly, in both *λ* and 24_B_ phages, deletion of the* exo-xis* region caused only moderate effects on expression of most genes from the S.O.S. regulon (Figure 7), contrary to hydrogen peroxide-treated bacteria where the differences were significantly higher (compare Figures 5 and 7). The exceptions in UV-irradiated cells were* rpoD, rpoH*, and* rpoS* genes in *λ* and* rpoH* and* rpoS* in 24_B_, whose expressions were at considerably lower level in the absence of the* exo-xis* region (Figure 7). One should also note that the induction of the S.O.S. regulon with UV irradiation was quicker than that with hydrogen peroxide. These results indicate that the influence of the* exo-xis* region on the S.O.S. response is particularly well pronounced under conditions of the oxidative stress.

**Figure 8:**
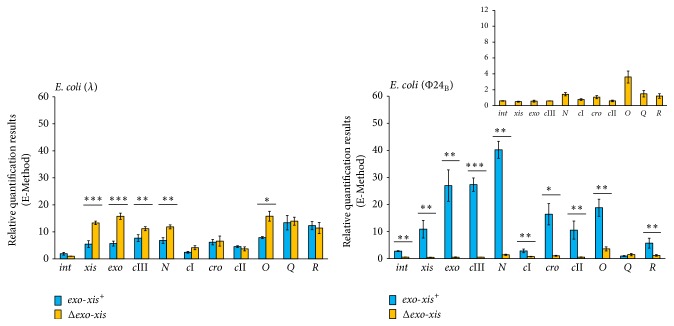
Expression of selected bacteriophage genes in* E. coli* MG1655 lysogenic with *λ* or Φ24_B_, either wild-type (blue columns) or Δ*exo-xis* (yellow columns) at 160 min after UV irradiation (50 J/m^2^), as estimated by reverse transcription quantitative real-time PCR. The values obtained with untreated cells were used as calibrators and were subtracted from the values determined at particular time points; thus, the presented values indicate the induction of expression of tested genes. The presented results are mean values from 3 independent experiments (biological samples), with error bars indicating SD. The additional panel for* E. coli* (24_B_) represents the results of Δ*exo-xis* variant with different scale, due to very small values measured. Statistically significant differences (in* t*-test) are marked as follows: ^*∗*^ *p* < 0.05, ^*∗∗*^ *p* < 0.01, and ^*∗∗∗*^ *p* < 0.001.

Unlike the S.O.S. regulon expression, levels of mRNAs of bacteriophage genes in UV-irradiated cells were affected similarly to those in hydrogen peroxide-treated lysogenic bacteria by the absence of the* exo-xis* region (Figure 8). Again, although some differences were observed between *λ* and *λ*Δ*exo-xis*, the differences between Φ24_B_ and Φ24_B_Δ*exo-xis* were dramatic. This indicates that the influence of the* exo-xis* region on expression of phage genes after prophage induction does not depend on the induction agent.

## 4. Conclusions

The* exo-xis* region is necessary for effective, RecA-dependent induction of Shiga toxin-converting bacteriophage Φ24_B_ under conditions of the oxidative stress. In hydrogen peroxide-treated* E. coli*, this region positively influences expression of the S.O.S. regulon in both Φ24_B_ and *λ* lysogens and expression of phage genes crucial for lytic development (particularly* xis, exo, N, cro, O, Q,* and* R*) in Φ24_B_, but not in *λ*. Since the oxidative stress appears to be the major cause of induction of Shiga toxin-converting prophages during infections of human intestine by enterohemorrhagic* E. coli* (EHEC), the* exo-xis* region and/or products of its expression might be considered as potential targets for anti-EHEC drugs.

## Figures and Tables

**Table 1:** *Escherichia coli* strains.

Strain	Genotype or relevant characteristics	Reference
*E. coli* MG1655	F^−^ *λ* ^−^ * ilvG rfb-50 rph-1*	[27]
*E. coli* MG1655 (*λ*)	MG1655 bearing *λ* prophage	[24]
*E. coli* MG1655 (*λ*Δ*exo-xis*)	MG1655 bearing *λ* prophage with deletion of all orfs and genes localized between genes *exo* and *xis*	This study, by recombination
*E. coli* MG1655 (*λ*Δ*orfs*)	MG1655 bearing *λ* prophage with deletion of *orf60a, orf63, orf61*, and *orf73*	This study, by recombination
*E. coli* MG1655 (*λ*Δ*orf60a*)	MG1655 bearing *λ* prophage with deletion of *orf60a *	This study, by recombination
*E. coli* MG1655 (*λ*Δ*orf63*)	MG1655 bearing *λ* prophage with deletion of *orf63 *	This study, by recombination
*E. coli* MG1655 (*λ*Δ*orf61*)	MG1655 bearing *λ* prophage with deletion of *orf61*	This study, by recombination
*E. coli* MG1655 (*λ*Δ*orf73*)	MG1655 bearing *λ* prophage with deletion of *orf73*	This study, by recombination
*E. coli* MG1655 (*λ*Δ*ea22*)	MG1655 bearing *λ* prophage with deletion of *ea22*	This study, by recombination
*E. coli* MG1655 (*λ*Δ*ea8.5*)	MG1655 bearing *λ* prophage with deletion of *ea8.5*	This study, by recombination
*E. coli* MG1655 (Φ24_B_)	MG1655 bearing Φ24_B_ prophage	[24]
*E. coli* MG1655 (Φ24_B_Δ*exo-xis*)	MG1655 bearing Φ24_B_ prophage with deletion of all orfs and genes localized between genes *exo* and *xis*	This study, by recombination
*E. coli* MG1655 (Φ24_B_Δ*orfs*)	MG1655 bearing Φ24_B_ prophage with deletion of 4 orfs being homologues of *λorf60a, λorf63, λorf61*, and *λorf73*	This study, by recombination
*E. coli* MG1655 (Φ24_B_Δ*orf60a*)	MG1655 bearing Φ24_B_ prophage with deletion of vb_24_B__9c, the homologue of *λorf60a *	This study, by recombination
*E. coli* MG1655 (Φ24_B_Δ*orf63*)	MG1655 bearing Φ24_B_ prophage with deletion of vb_24_B__8c, the homologue of *λorf63 *	This study, by recombination
*E. coli* MG1655 (Φ24_B_Δ*orf61*)	MG1655 bearing Φ24_B_ prophage with deletion of vb_24_B__7c, the homologue of *λorf61*	This study, by recombination
*E. coli* MG1655 (Φ24_B_Δ*orf73*)	MG1655 bearing Φ24_B_ prophage with deletion of the sequence of putative C4 zinc finger protein, the homologue of *λorf73*	This study, by recombination
*E. coli* MG1655 (Φ24_B_Δ*ea22*)	MG1655 bearing Φ24_B_ prophage with deletion of vb_24_B__6c, the analogue of *λea22*	This study, by recombination
*E. coli* MG1655*recA13*	MG1655 but *recA13*	[33]
*E. coli* MG1655*recA13 *(*λ*)	MG1655*recA13* bearing *λ* prophage	This study, by lysogenization
*E. coli* MG1655*recA13 *(*λ*Δ*exo-xis*)	MG1655*recA13* bearing *λ* prophage with deletion of all orfs and genes localized between genes *exo* and *xis*	This study, by lysogenization
*E. coli* MG1655*recA13 *(Φ24_B_)	MG1655*recA13* bearing Φ24_B_ prophage	This study, by lysogenization
*E. coli* MG1655*recA13 *(Φ24_B_Δ*exo-xis*)	MG1655*recA13* bearing Φ24_B_ prophage with deletion of all orfs and genes localized between genes *exo* and *xis*	This study, by lysogenization
*E. coli *PQ37	*sfi*A::Mud(Ap *lac*) *cts*, *lac*ΔU169, *mal* ^+^, *gal*E, *gal*Y, PhoC, *rfa*, F^−^, *thr*, *leu*, *his*, *pyrD*, *thi*, *trp*::*MUC* ^+^, *srl300*::Tn*10*, *rpoB*, *uvrA* ^+^	[31]
*E. coli *PQ37 (*λ*)	PQ37 bearing *λ* prophage	This study, by lysogenization
*E. coli *PQ37 (*λ*Δ*exo-xis*)	PQ37 bearing *λ* prophage with deletion of all orfs and genes localized between genes *exo* and *xis*	This study, by lysogenization
*E. coli *PQ37 (Φ24_B_)	PQ37 bearing Φ24_B_ prophage	This study, by lysogenization
*E. coli *PQ37 (Φ24_B_Δ*exo-xis*)	PQ37 bearing Φ24_B_ prophage with deletion of all orfs and genes localized between genes *exo* and *xis*	This study, by lysogenization

**Table 2:** Primers used for construction of *E. coli *strains.

Primer name	Sequence (5′→3′)
pF_*λ*_exo-xis = pF_*λ*_orf60a pR_*λ*_exo-xis	ATATCCGGGTAGGCGCAATCACTTTCGTCTACTCCGTTACAAAGCGAGGAATTAACCCTCACTAAAGGGCG AGGCGGCTTGGTGTTCTTTCAGTTCTTCAATTCGAATATTGGTTACGTCTTAATACGACTCACTATAGGGCTC

pF_*λ*_orfs = pF_*λ*_orf60a pR_*λ*_orfs = pR_*λ*_orf73	ATATCCGGGTAGGCGCAATCACTTTCGTCTACTCCGTTACAAAGCGAGGAATTAACCCTCACTAAAGGGCG ACCTCTCTGTTTACTGATAAGTTCCAGATCCTCCTGGCAACTTGCACAAGTAATACGACTCACTATAGGGCTC

pF_*λ*_orf60a pR_*λ*_orf60a	ATATCCGGGTAGGCGCAATCACTTTCGTCTACTCCGTTACAAAGCGAGGAATTAACCCTCACTAAAGGGCG AGATGCTTTGTGCATACAGCCCCTCGTTTATTATTTATCTCCTCAGCCAGTAATACGACTCACTATAGGGCTC

pF_*λ*_orf63 pR_*λ*_orf63	CACAAAGCATCTTCTGTTGAGTTAAGAACGAGTATCGAGATGGCACATAGAATTAACCCTCACTAAAGGGCG TCATACCTGGTTTCTCTCATCTGCTTCTGCTTTCGCCACCATCATTTCCATAATACGACTCACTATAGGGCTC

pF_*λ*_orf61 pR_*λ*_orf61	AGAGAAACCAGGTATGACAACCACGGAATGCATTTTTCTGGCAGCGGGCTAATTAACCCTCACTAAAGGGCG TTATCCGGAAACTGCTGTCTGGCTTTTTTTGATTTCAGAATTAGCCTGACTAATACGACTCACTATAGGGCTC

pF_*λ*_orf73 pR_*λ*_orf73	ACATCATTGATTCAGCATCAGAAATAGAAGAATTACAGCGCAACACAGCAAATTAACCCTCACTAAAGGGCG ACCTCTCTGTTTACTGATAAGTTCCAGATCCTCCTGGCAACTTGCACAAGTAATACGACTCACTATAGGGCTC

pF_*λ*_ea22 pR_*λ*_ea22	GAAATTAACTCTCAGGCACTGCGTGAAGCGGCAGAGCAGGCAATGCATGAAATTAACCCTCACTAAAGGGCG GTCAGACATCATATGCAGATACTCACCTGCATCCTGAACCCATTGACCTCCTAATACGACTCACTATAGGGCTC

pF_*λ*_ea8.5 pR_*λ*_ea8.5	ATGAGTATCAATGAGTTAGAGTCTGAGCAAAAAGATTGGGCGTTATCAATAATTAACCCTCACTAAAGGGCG TAATCATCTATATGTTTTGTACAGAGAGGGCAAGTATCGTTTCCACCGTATAATACGACTCACTATAGGGCTC

pF_Φ24_B__exo-xis = pF_24_B__orf60a pR_Φ24_B__exo-xis	TGGTAATGAAGCATCCTCACGATAATATCCGGGTAGGCACGATCACTTTCAATTACCTCACTAAAGGGCG TGGCAATATGCTTTTCCTTCTCAATTTCCGCTTTAATCATATGCAGTTCGTAATACGACTCACTATAGGGCTC

pF_Φ24_B__orfs = pF_Φ24_B__orf60a pR_Φ24_B__orfs = pR_Φ24_B__orf73	TGGTAATGAAGCATCCTCACGATAATATCCGGGTAGGCACGATCACTTTCAATTACCTCACTAAAGGGCG CTTCGAACCTCTCTGTTTACTGATAAGCTCCAGATCCTCCTGGCAACTTGTAATACGACTCACTATAGGGCTC

pF_Φ24_B__orf60a pR_Φ24_B__orf60a	TGGTAATGAAGCATCCTCACGATAATATCCGGGTAGGCACGATCACTTTCAATTACCTCACTAAAGGGCG GGAGATGCTTTGTGCATACAGCCCCTCGTTATTATTTATCTCTTCAGCCTAATACGACTCACTATAGGGCTC

pF_Φ24_B__orf63 pR_Φ24_B__orf63	TGCACAAAGCATCTCCTGTTGAATTAAGAACGAGTATCGGGATGGCACATAATTAACCCTCACTAAAGGGCG CATCATTTCCAGCTTTTGTGAAAGGGATGTGGCTAACGTATGAAATTCTTTAATACGACTCACTATAGGGCTC

pF_Φ24_B__orf61 pR_Φ24_B__orf61	TTCTGGCAGCAGGCTTCATATTCTGTGTGCTTATGCTTGCCGACATGGGAAATTAACCCTCACTAAAGGGCG CACCGTTCCTTAAAGACGCCGTTTAACATGCCGATCGCCAGACTTAAATGTACGACTCACTATAGGGCTC

pF_Φ24_B__orf73 pR_Φ24_B__orf73	TGGCAGACCTCATTGATTCAGCATCAGAAATTGAAGAATTACAGCGCAACAATTAACCCTCACTAAAGGGCGG CTTCGAACCTCTCTGTTTACTGATAAGCTCCAGATCCTCCTGGCAACTTGTAATACGACTCACTATAGGGCTC

pF_Φ24_B__ea22 pR_Φ24_B__ea22	ATCAGGCACTGCGTGAAAAGGCAGAGAAAGCAACTAAAGGAAGCTACATCAATTAACCCTCACTAAAGGGCG ATCCAGTGTGACGGTTTCCACGACGCACCAGGAATTATCCACCCATCATTATACGACTCACTATAGGGCTC

**Table 3:** Primers used for PCR.

Primers for bacterial sequences		Primers for phage sequences	
Name	Sequence (5′→3′)	Name	Sequence (5′→3′)
pF_recA	AGATTTCGACGATACGGCCC	pF_*λ*_int	TTTGATTTCAATTTTGTCCCACT
pR_recA	AACCATCTCTACCGGTTCGC	pR_ *λ*_int	ACCATGGCATCACAGTATCG
pF_lexA	ATGGATGGTGACTTGCTGGC	pF_*λ*_xis	TACCGCTGATTCGTGGAACA
pR_lexA	TTCGTCATCAATACGTGCGAC	pR_ *λ*_xis	GGGTTCGGGAATGCAGGATA
pF_ssb	ATCGAAGGTCAGCTGCGTAC	pF_*λ*_exo	TGCCGTCACTGCATAAACC
pR_ssb	CGACTTCTGTGGTGTAGCGA	pR_ *λ*_exo	TCTATCGCGACGAAAGTATGC
pF_recF	CGATACCGGCGCTATACTCC	pF_*λ*_cIII	ATTCTTTGGGACTCCTGGCTG
pR_recF	TTACGAACAGCTACGCCCG	pR_ *λ*_cIII	GTAAATTACGTGACGGATGGAAAC
pF_rpoD	GAATCTGAAATCGGGCGCAC	pF_*λ*_N	CTCGTGATTTCGGTTTGCGA
pR_rpoD	GTCAACAGTTCAACGGTGCC	pR_ *λ*_N	AAGCAGCAAATCCCCTGTTG
pF_rpoH	GCTTTGGTGGTCGCAACTTT	pF_*λ*_cI	ACCTCAAGCCAGAATGCAGA
pR_rpoH	TCGCCGTTCACTGGATCAAA	pR_ *λ*_cI	CCAAAGGTGATGCGGAGAGA
pF_rpoS	TTGCTCTGCGATCTCTTCCG	pF_*λ*_cro	ATGCGGAAGAGGTAAAGCCC
pR_rpoS	GAACGTTTACCTGCGAACCG	pR_ *λ*_cro	TGGAATGTGTAAGAGCGGGG
pF_uvrA	GTCCATATCCGCCACTACCG	pF_*λ*_cII	TCGCAATGCTTGGAACTGAGA
pR_uvrA	TTACCCAACGTCTTGCCGAG	pR_ *λ*_cII	CCCTCTTCCACCTGCTGATC
pF_ftsK	ACAAACCGTTTATCTGCGCG	pF_*λ*_O	AATTCTGGCGAATCCTCTGA
pR_ftsK	ATCTTTACCCAGCACCACGG	pR_ *λ*_O	GAATTGCATCCGGTTT
pF_16SrRNA	CCTTACGACCAGGGCTACAC	pF_*λ*_Q	TTCTGCGGTAAGCACGAAC
pR_16SrRNA	TTATGAGGTCCGCTTGCTCT	pR_ *λ*_Q	TGCATCAGATAGTTGATAGCCTTT
** **	** **	pF_*λ*_R	ATCGACCGTTGCAGCAATA
** **	** **	pR_ *λ*_R	GCTCGAACTGACCATAACCAG
** **	** **	pF_Φ24_B__int	CAGTTGCCGGTATCCCTGT
** **	** **	pR_ Φ24_B__int	TGAGGCTTTCTTGCTTGTCA
		pF_Φ24_B__xis	TATCGCGCCGGATGAGTAAG
		pR_ Φ24_B__xis	CGCACAGCTTTGTATAATTTGCG
		pF_Φ24_B__exo	TGCCGTCACTGCATAAACC
		pR_ Φ24_B__exo	TCTATCGCGACGAAAGTATGC
** **	** **	pF_Φ24_B__cIII	ATTCTTTGGGACTCCTGGCTG
** **	** **	pR_ Φ24_B__cIII	GTAAATTACGTGACGGATGGAAAC
** **	** **	pF_Φ24_B__N	AGGCGTTTCGTGAGTACCTT
		pR_ Φ24_B__N	TTACACCGCCCTACTCTAAGC
** **	** **	pF_Φ24_B__cI	TGCTGTCTCCTTTCACACGA
** **	** **	pR_ Φ24_B__cI	GCGATGGGTGGCTCAAAATT
** **	** **	pF_Φ24_B__cro	CGAAGGCTTGTGGAGTTAGC
** **	** **	pR_ Φ24_B__cro	GTCTTAGGGAGGAAGCCGTT
** **	** **	pF_Φ24_B__cII	TGATCGCGCAGAAACTGATTTAC
** **	** **	pR_ Φ24_B__cII	GACAGCCAATCATCTTTGCCA
** **	** **	pF_Φ24_B__O	AAGCGAGTTTGCCACGAT
** **	** **	pR_ Φ24_B__O	GAACCCGAACTGCTTACCG
		pF_Φ24_B__Q	GGGAGTGAGGCTTGAGATGG
		pR_ Φ24_B__Q	TACAGAGGTTCTCCCTCCCG
		pF_Φ24_B__R	GGGTGGATGGTAAGCCTGT
		pR_ Φ24_B__R	TAACCCGGTCGCATTTTTC

**Table 4:** Duration of the lag phase of the phage lytic development after prophage induction with either hydrogen peroxide (1 mM) or UV irradiation (50 J/m^2^).

Strain	The time range of the switch from lag to log phase	
	H_2_O_2_ (1 mM)	UV (50 J/m^2^)
MG1655 (*λ*)	60–90 min	60 min
MG1655 (*λ*Δ*exo-xis*)	90–120 min	30–60 min
MG1655 (*λ*Δ*orfs*)	60–90 min	30–60 min
MG1655 (*λ*Δ*orf60a*)	60–90 min	30–60 min
MG1655 (*λ*Δ*orf63*)	60–90 min	30–90 min
MG1655 (*λ*Δ*orf61*)	60–90 min	30–60 min
MG1655 (*λ*Δ*orf73*)	30–60 min	0–30 min
MG1655 (*λ*Δ*ea22*)	60–90 min	30–60 min
MG1655 (*λ*Δ*ea8.5*)	60–90 min	30–60 min
MG1655 (Φ24_B_)	60–90 min	150–180 min
MG1655 (Φ24_B_Δ*exo-xis*)	a	150–180 min
MG1655 (Φ24_B_Δ*orfs*)	150–180 min	150–180 min
MG1655 (Φ24_B_Δ*orf60a*)	120–150 min	90–120 min
MG1655 (Φ24_B_Δ*orf63*)	120–150 min	30–60 min
MG1655 (Φ24_B_Δ*orf61*)	90–120 min	30–60 min
MG1655 (Φ24_B_Δ*orf73*)	90–120 min	120–150 min
MG1655 (Φ24_B_Δ*ea22*)	120–150 min	30–60 min

^a^The value was not determined due to a very low efficiency of prophage induction under these conditions (as shown in Figure 2).
